# Matching Rotator Cuff Repair Construct to Tear Pattern to Achieve Anatomic Restoration and Minimize Tension Mismatch for Repairable Posterosuperior Tears

**DOI:** 10.1016/j.eats.2025.103553

**Published:** 2025-07-05

**Authors:** Mustafa Saad Rashid, Georgios Mamarelis, Michele Yong Ling Ding Novak, Ian K.Y. Lo

**Affiliations:** a East Suffolk and North Essex NHS Foundation Trust, Colchester, England; b King’s College Hospital NHS Foundation Trust, London, England; c Section of Sport and Exercise Medicine, Department of Family Medicine, University of Calgary, Calgary, Canada; d Section of Orthopedics, Department of Surgery, University of Calgary, Calgary, Canada

## Abstract

Rotator cuff tears are all unique, with certain patterns that can categorize certain types of tears. This article focuses on exploring how tear pattern, tear mobility, and the tear reduction vector can be assessed arthroscopically to provide surgeons with a framework for using the most appropriate construct for each tear. This surgical decision-making framework aims to empower surgeons to look beyond repairing all tears with the same construct and, rather, introduce some nuance in matching the repair construct to the tear, thereby enabling better anatomic restoration and minimizing tension mismatch.

The management of posterosuperior rotator cuff tears has seen significant innovation regarding suture anchors, suture types, surgical techniques, and augmentation strategies.^1^, ^2^, ^3^, ^4^, ^5^, ^6^ However, achieving successful healing remains a challenge.^7^^,^^8^ For some time, surgeons have fervently debated the virtues of single- versus double-row repair constructs.^9^, ^10^, ^11^ Instead of thinking of the optimal construct in isolation, we prefer to consider the “most appropriate construct” (MAC) with respect to tear size, tear mobility, and the tear reduction vector. After considering these factors intraoperatively, we use the MAC.

The surgeon not only must be agile and capable of assessing these factors intraoperatively but also must able to perform a variety of repair constructs. This article will outline this concept in detail. The facets of this philosophy are intuitive to most surgeons and largely supported by biomechanical evidence. The MAC paradigm relates to matching the repair construct to the tear pattern and tear reduction vector. At its pinnacle, the goal is to achieve an anatomic or near anatomic repair, when possible. This article focuses on the key steps that allow the treating surgeon to use the MAC philosophy to address posterosuperior cuff repairs.

## Surgical Technique

### Positioning, Diagnostic Arthroscopy, and Addressing of Anterior Structures

**Fig 1:**
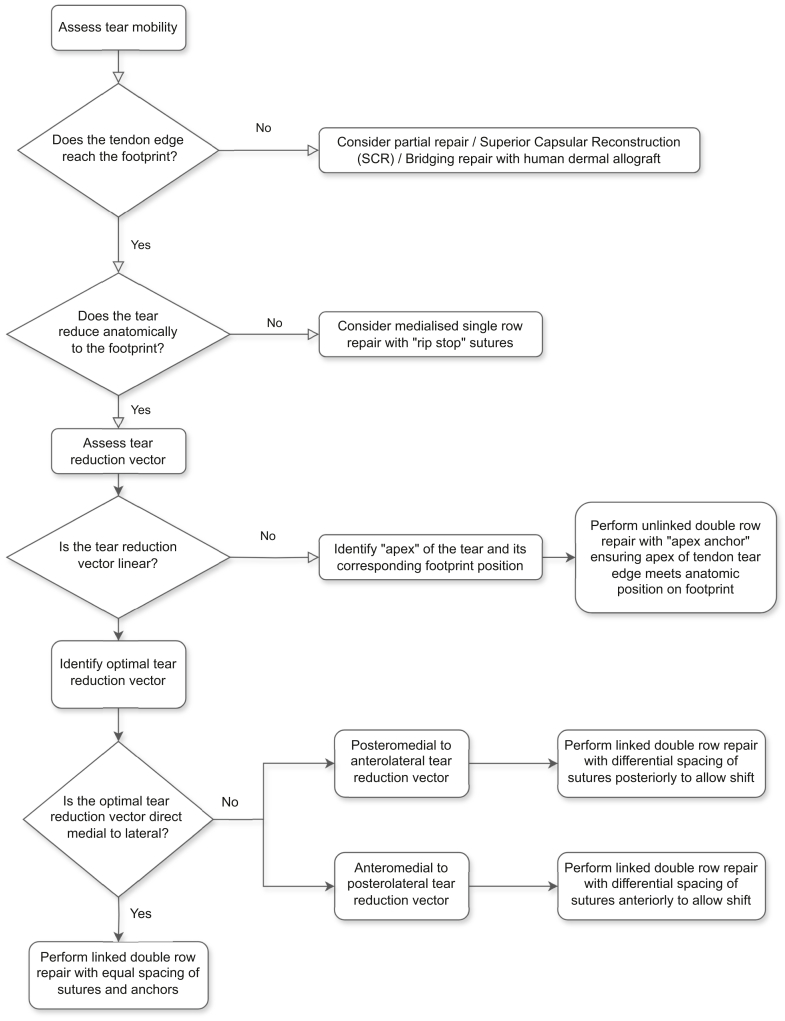
Flowchart showing the steps to consider when choosing the most appropriate construct for posterosuperior cuff tear repair. (SCR, superior capsular reconstruction.)

With the patient placed in the lateral decubitus position under general anesthesia, a posterior viewing portal is established (Video 1). Starting with a 30° arthroscope, a diagnostic arthroscopy is performed in a systematic fashion, starting with the anterior structures including the subscapularis tendon, as well as the long head of the biceps (LHB) tendon. If there is suspicion that the LHB and/or subscapularis tendon is pathologic, then a biceps tenodesis is performed. This is often performed using a 70° arthroscope, releasing the transverse humeral ligament with a radiofrequency device, and performing an in situ tenodesis using a knotless anchor in the lower part of the groove, prior to excising the intra-articular portion of the LHB tendon. This portion of the technique is standard. The steps that define the MAC philosophy are described in the next sections. Figure 1 outlines a flowchart that describes the key components of this treatment paradigm.

### Tear Assessment and Footprint Preparation of Small Tears

Assessment of the supraspinatus is performed from the intra-articular view using the posterior portal and a 70° arthroscope. In smaller tears, an anterosuperolateral (ASL) portal is established via an outside-in technique using a spinal needle. This becomes the working portal, and a radiofrequency ablation device is used to denude the footprint of soft tissue. The footprint is then prepared with an elliptical burr in reverse mode to lightly decorticate the bony bed. The posterior viewing portal is now switched from an intra-articular view to a subacromial view. In all tears, the subacromial bursa is debrided using an oscillating soft-tissue shaver from the ASL portal. By switching back to a 30° arthroscope and viewing from the ASL portal, the shaver can be inserted via the posterior portal and the posterolateral subacromial bursa is debrided.

### Performing of Releases in Larger Tears

In larger tears, releases may be necessary. We do not advocate interval slides because it is believed that it is best to keep the cuff in continuity where possible. Instead, a radiofrequency device is inserted via the posterior portal (viewing from the ASL portal), and the underside of the acromion is skeletonized. This creates a “curtain” of bursa and rotator cuff tendon. Careful shaving of the bursa will then reveal the underlying tendon. At the end of these releases, the spine of the scapula will be visible. It may also be necessary to perform releases on the underside of the rotator cuff. This is best achieved using a 70° arthroscope, viewing from the posterior portal, and a Bankart elevator is brought in from the ASL portal underneath the tendon and slid along the glenoid neck superiorly and posterosuperiorly.

### Testing of Tear Mobility and Reduction Vector

**Fig 2:**
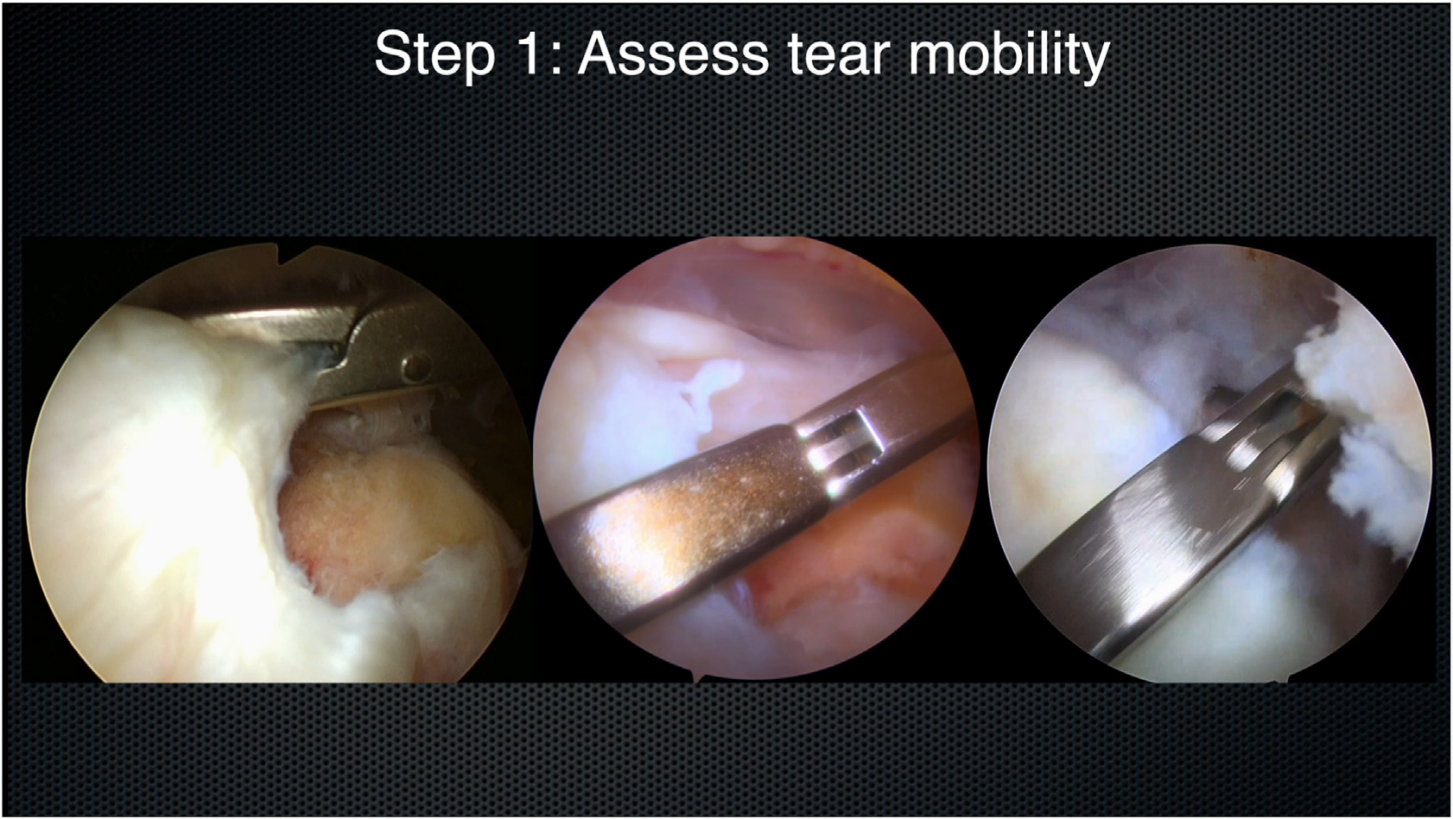
Step 1: assess tear mobility. Viewing from the posterior portal (right shoulder) with a 70° arthroscope, a tendon grasper is placed on the tear edge from the anterosuperolateral portal. The tendon edge is tested for mobility.

**Fig 3:**
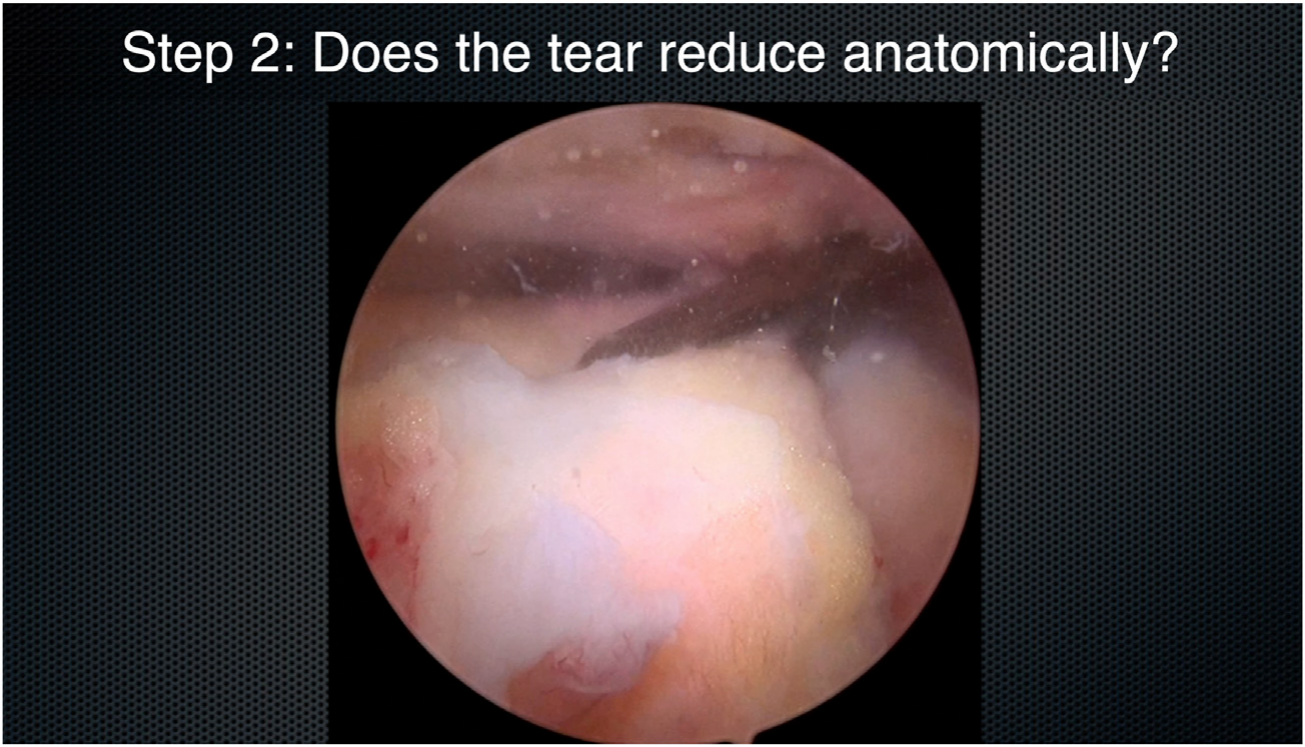
Step 2: determine whether the tear reduces anatomically. Viewing from the posterior portal (right shoulder) with a 70° arthroscope, the tendon edge is reduced using the tendon grasper from the superolateral portal to cover the footprint.

**Fig 4:**
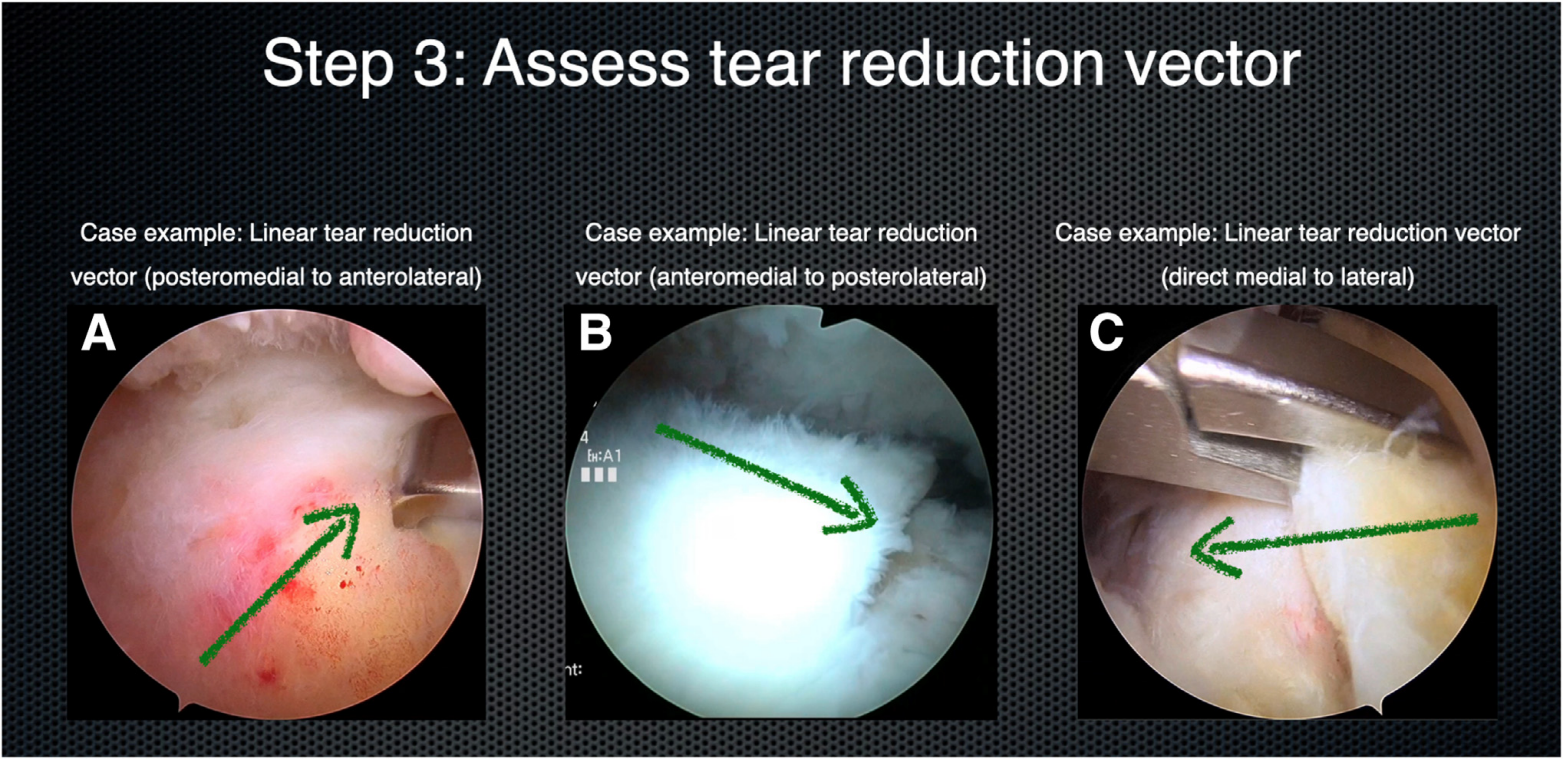
Step 3: assess the tear reduction vector. (A) The tendon grasper reduces the tear in the posteromedial-to-anterolateral direction (arrow) (right shoulder, viewing from the posterior portal). (B) The tendon grasper reduces the tear in the anteromedial-to-posterolateral direction (arrow) (right shoulder, viewing from the posterior portal). (C) The tendon grasper reduces the tear in the direct medial-to-lateral direction (arrow) (left shoulder, viewing from the posterior portal).

Step 1 of using the MAC philosophy is to assess tear mobility (Fig 2). The tear is then reassessed from the subacromial perspective, paying close attention to the tear size and pattern (crescent, L shaped, or reverse L shaped). A tendon grasper is brought in from the ASL portal, and various locations of the tendon edge are grasped and brought to various locations of the footprint. This maneuver allows the surgeon to appreciate whether the tear will reduce anatomically (Fig 3) and to assess the tear reduction vector and tear mobility. The surgeon should test different areas of the tendon edge by grasping the tissue at different locations and pulling it over to different points on the bony footprint. The surgeon should start in a direct medial-to-lateral direction, before assessing anteromedial-to-posterolateral and posteromedial-to-anterolateral directions. The surgeon should test different areas of the tendon edge—anterior and posterior. This step is key to generate a 3-dimensional representation of tear mobility and the tear reduction vector. Occasionally, the tear reduction vector is from directly medial to directly lateral. More commonly, it is from posteromedial to anterolateral. Occasionally, the tear is best reduced from an anteromedial-to-posterolateral direction (Fig 4). Rarely, for more complex tear patterns, the tendon edge may need to “turn a corner” to achieve an anatomic reduction.

### Nonanatomic Repair for Larger, Immobile Tears

After careful assessment, the surgeon may find that the tear type, pattern, or mobility does not permit an anatomic reduction. If the tendon edge can be brought to the medial aspect of the footprint, the surgeon may consider a nonanatomic repair. In this scenario, we recommend medializing the footprint (5-8 mm), placing rip-stop sutures (typically 2-4 horizontal mattress sutures), and performing a single-row repair. If the tendon edge does not reach the medial footprint after surgical releases have been performed, then the surgeon has various nonanatomic options available. Options include partial repair, superior capsular reconstruction, bridging repair with human dermal allograft, and lower trapezius tendon transfer. These nonanatomic techniques will not be discussed in detail in this article.

### Determination of Which Construct Is Most Appropriate

**Fig 5:**
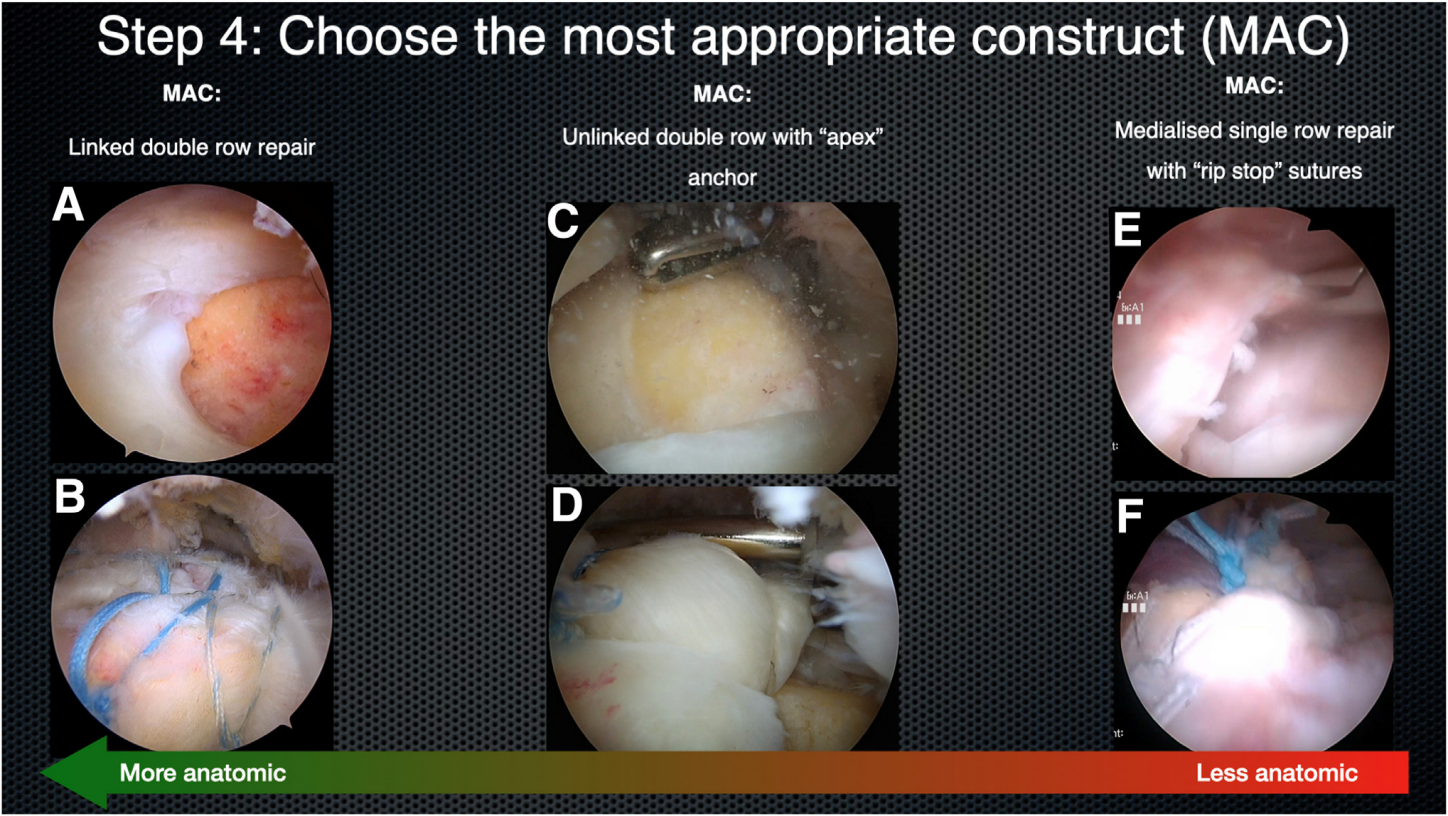
Step 4: choose the most appropriate construct (MAC). (A, B) A mobile tear that reduces anatomically is repaired with a linked double-row construct (right shoulder, viewing from the posterior portal). (C, D) A complex tear that reduces in a nonlinear fashion is repaired with an unlinked double-row construct using an apex anchor technique (right shoulder, viewing from the posterior portal). (E, F) A less mobile tear that does not reduce anatomically is repaired with a medialized single-row construct with rip-stop sutures (right shoulder, viewing from the posterior portal). These constructs vary from more anatomic (left) to less anatomic (right) as shown by the arrow.

Next, the surgeon will decide which repair construct is most appropriate based on tear mobility, tear pattern, tendon quality, and the tear reduction vector (Fig 5). Relevant considerations include the following: Is an anatomic repair possible? If so, is the tear sufficiently mobile to reach the bony footprint without much tension? What is the direction of the tear reduction vector? If oblique, what position is optimal for placing the sutures from the anteromedial and posteromedial anchors? If an anatomic repair is not possible, are all the subacromial and supraglenoid releases complete? Does the tendon edge reach the medial portion of the footprint without tension? If so, then a medialized single-row repair may be most appropriate. If the tendon edge is insufficiently mobile to reach the medial footprint, is the remaining stump robust enough to hold sutures and to secure a bridging graft? If a nonanatomic repair is chosen, can the posterior cuff (infraspinatus and teres minor) be repaired?

In all repairs, the same footprint preparation technique is used. Debridement of the bony footprint is performed using a radiofrequency ablation device, followed by light decortication with an arthroscopic burr (in reverse mode).

### Linked Double-Row Repair

A linked double-row repair may be most appropriate when the tear is mobile and can be reduced anatomically without tension and the tear reduction vector is linear. Medial-row anchors (1-3 depending on tear size) are placed. If the tear reduction vector is directly medial to lateral, sutures are passed using a suture passing device (Scorpion; Arthrex, Naples, FL) from anterior to posterior with equal spacing to allow secure fixation to the entire tendon edge. If the tear reduction vector is oblique but linear, the suture passage should be spaced within the tendon to compensate for this. For example, if the tear requires an anteromedial-to-posterolateral reduction vector, placing the sutures from the medial-row anchors more anteriorly will adjust the direction of pull. If the tear requires a posteromedial-to-anterolateral reduction vector, placing the sutures from the medial-row anchors more posteriorly will assist in achieving anatomic footprint restoration. The sutures are tied in a horizontal mattress configuration, and one or both limbs from each pair are brought into a knotless lateral-row anchor. The limbs are then cut after securing the lateral row.

### Unlinked Double-Row Repair With Apex Anchor

If the tear reduction vector is nonlinear, typically with an L-shaped path of the tendon edge, the “apex” of the tendon edge should identified. The surgeon must determine where this area of the tendon should be positioned on the bony footprint to “key in” the tear into an antomic position. Medial-row anchors are placed (typically 2 double-loaded anchors are needed), and the sutures from these anchors are passed into the tendon, ensuring appropriate spacing between sutures, but they are not tied. The apex anchor is inserted into the lateral row at the point at which the identified tendon apex needs to meet the bony footprint for an anatomic repair. The sutures from this anchor are passed into the apex of the tendon tear (typically in a simple suture configuration). These sutures are tied first, followed by the medial-row sutures, to compress the entire footprint. Finally, some suture limbs from the medial-row anchors are selected to bring into a knotless lateral-row anchor to complete the repair.

### Medialized Single-Row Repair With Rip-Stop Sutures

If, after testing of tear mobility and the tear reduction vector, the tear is incapable of being repaired anatomically without undue tension, then a medialized single-row repair may be most appropriate. To increase tendon security, 2 to 4 free horizontal mattress sutures are placed, tied, and cut to act as rip-stop sutures. The footprint is medialized by 5 to 8 mm using a radiofrequency ablation device and burr (in reverse mode). Two to three suture anchors are inserted into the new footprint. By use of a suture passing device (Scorpion), one limb from each pair is passed medial to the rip-stop sutures. Each pair of sutures is tied and then cut.

## Discussion

This surgical paradigm allows the reader to reframe the discussion surrounding single- versus double-row repair. Rather than being confined to treating each tear with either a single- or double-row repair, the reader can now use the steps outlined in the MAC philosophy to repair each tear with a nuance that maximizes the chances of success by minimizing undue tension. Our approach offers flexibility in how to approach repairing each tear and allows the subtle distinctions between tears to be appreciated. Assessing tear type, pattern, and mobility and the tear reduction vector is key to adopting the MAC technique. Arthroscopic knot-tying proficiency is essential for ensuring stable constructs and adequate compression of the tendon to the bony footprint. Pearls and pitfalls of the MAC philosophy are presented in Table 1, and advantages and disadvantages are presented in Table 2.

**Table 1:** Pearls and Pitfalls of Most Appropriate Construct Philosophy

Pearls
Use a 70° arthroscope as well as a 30° arthroscope to achieve a new and wider perspective of the cuff tear.
Clear the posterolateral subacromial bursa using an arthroscopic oscillating shaver from the posterior portal (view from the anterosuperolateral or lateral portal).
Assess all tears by viewing from the posterior portal using a 70° arthroscope and testing tear mobility and the tear reduction vector using a grasper placed at multiple locations on the tendon edge.
Prepare the footprint using a radiofrequency ablation device, followed by an elliptical burr in reverse mode.
Place every anchor through an independent accessory lateral portal to aid in suture management.
Pitfalls
Bear in mind that determining when tear mobility does not permit anatomic reduction can be challenging and takes practice to master.
Note that it is often difficult to assess nonlinear tear reduction vectors using a grasper from the anterosuperolateral or lateral portal.
When using rip-stop sutures, ensure that the simple sutures are passed medial to these. Make sure you leave sufficient space medial to the rip-stop sutures to allow suture passage.
When using an unlinked double-row construct, ensure that you have clearly identified where the apex of the tendon edge is and to which corresponding area this part of the tendon reduces.

**Table 2:** Advantages and Disadvantages of Most Appropriate Construct Philosophy

Advantages
More anatomic reduction of the cuff tear will minimize any tension mismatch across the repair construct.
Avoiding bringing larger, less mobile tears into a linked double-row construct will avoid strangulation and potentially type 2 (medial) cuff repair failures.
Application of this philosophy for treating rotator cuff tears introduces nuance and allows the surgeon to better appreciate the subtleties of individual tears.
Disadvantages
Assessing tear size, pattern, and mobility and the tear reduction vector adds surgical time.
Using a variety of repair constructs requires a variety of anchor types, as well as astute surgical assistants to ensure the wider surgical team works efficiently.
It is impossible to effectively deploy the various repair constructs described without proficient arthroscopic knot tying.

Surgeons wishing to incorporate the MAC technique into their practice should begin by gaining experience in testing tear mobility and assessing the tear reduction vector. If the tear remains immobile, the surgeon should first consider whether all releases have been completed in their entirety. After mobilization of the tear, the following 4 steps are helpful: (1) Assess tear mobility (i.e., does the tear reach the footprint?). (2) Determine whether the tear is reduced to the footprint anatomically. (3) Assess the tear reduction vector (i.e., is it linear or non-linear, and what direction to the tear best reduce to the footprint?). (4) Match the construct to tear pattern, tear mobility, and the tear reduction vector.

This article outlines our framework for considering which construct to use when repairing a full-thickness rotator cuff tear. Surgeons should consider the MAC for each individual tear. Mobile tears without tendon loss may be repaired using a linked double-row repair, whereas less mobile and retracted tears may be most appropriately repaired with a medialized single-row repair with rip-stop sutures. Complex tear types with nonlinear tear reduction vectors may benefit from an unlinked double-row repair using an apex anchor technique.

## Disclosures

The authors declare the following financial interests/personal relationships which may be considered as potential competing interests: I.K.Y.L. reports a consulting or advisory relationship with Smith & Nephew and Wolters Kluwer Health; receives funding grants from Smith & Nephew; receives speaking and lecture fees from Smith & Nephew; receives travel reimbursement from Smith & Nephew; has a patent with royalties paid to Smith & Nephew; and has a patent with royalties paid to Arthrex. All other authors (M.S.R., G.M., M.Y.L.D.N.) declare that they have no known competing financial interests or personal relationships that could have appeared to influence the work reported in this paper.

## Supplementary Data

Video 1 Key components of the most appropriate construct (MAC) treatment philosophy for posterosuperior rotator cuff tears. Key steps of the surgical technique are included, describing how to use the MAC for repairing posterosuperior rotator cuff tears. In the video there are multiple examples of patients undergoing rotator cuff repair to both right and left shoulders. However, all patients are in the lateral decubitus position, viewing from the posterior portal with a 70∗ arthroscope.
